# A novel papillomavirus in a New Zealand fur seal (Arctocephalus forsteri) with oral lesions

**DOI:** 10.1038/s44298-024-00020-w

**Published:** 2024-03-21

**Authors:** Jonathon C. O. Mifsud, Jane Hall, Kate Van Brussel, Karrie Rose, Rhys H. Parry, Edward C. Holmes, Erin Harvey

**Affiliations:** 1 Sydney Institute for Infectious Diseases, School of Medical Sciences, The University of Sydney, Sydney, NSW 2006 Australia; 2 Centre for Planetary Health and Food Security, School of Environment and Science, Griffith University, Nathan, QLD Australia; 3 Australian Registry of Wildlife Health, Taronga Conservation Society Australia, Mosman, NSW 2088 Australia; 4 School of Chemistry and Molecular Biosciences, The University of Queensland, Brisbane, QLD 4067 Australia

**Keywords:** Virology, Metagenomics

## Abstract

Despite being the predominant seal species in the Australian-New Zealand region and serving as a key indicator of marine environmental health, little is known about infectious diseases in New Zealand fur seals (Long-nosed fur seal; *Arctocephalus forsteri*). Several papillomaviruses have been identified in earless seals and sea lions, with the latter linked to cutaneous plaques and invasive squamous cell carcinoma. To date, no papillomaviruses have been reported in fur seals. We used traditional veterinary diagnostic techniques and metatranscriptomic sequencing of tissue samples to investigate the virome of New Zealand fur seals. We identified a novel papillomavirus, provisionally termed *A. forsteri* papillomavirus 1 (AforPV1) in an animal with clinically and histologically identified oral papilloma-like lesions. RT-PCR confirmed the presence of AforPV1 only in oral papilloma samples from the affected individual. Phylogenetic analysis of the complete 7926 bp genome of AforPV1 revealed that it grouped with taupapillomaviruses found in related Carnivora species. These findings highlight the need for further research into the disease associations and impact of undiagnosed and novel viruses on New Zealand fur seals.

## Introduction

New Zealand fur seals (Long-nosed fur seal; *Arctocephalus forsteri*) are the most abundant seal species in the Australian-New Zealand region^1^. Their breeding colonies span the southern coast of Australia, ranging from Western Australia to Tasmania, as well as the coastlines and offshore islands of New Zealand and its subantarctic islands^2^. As long-lived marine mammals that feed at high trophic levels, New Zealand fur seals serve as important marine sentinels and offer insights into the health of aquatic ecosystems^3^.

Despite their importance, our understanding of infectious diseases in New Zealand fur seals, particularly those due to viruses, remains limited. A single circovirus has been identified from a New Zealand fur seal fecal sample^4^, although no details on the health status of the seal or the potential for pathogenicity were provided. The impact viruses can have on other seal species is well established, with numerous viral diseases described, including urogenital carcinoma associated with Otarine herpesvirus 1 in California sea lions (*Zalophus californianus*) and a South American fur seal (*A. australis*)^5,6^ and vesicular exanthema associated with San Miguel sea lion virus reported in sea lions, fur seals, and elephant seals along the western coast of the United States^7^. Viral diseases in seals can have far-reaching consequences^8^. For instance, outbreaks of seal influenza A (H10N7) across Europe in 2014^9^ and highly pathogenic avian influenza A (H5N1) in New England, USA, in 2023, both caused mass mortalities of harbor seals (*Phoca vitulina*)^10^. Additionally, seals may have the potential to affect human health through zoonotic virus transmission, particularly during close interactions such as stranding and mass mortality events^11^.

The *Papillomaviridae* are a family of non-enveloped, double-stranded DNA viruses, typically 7500 bp in length, that exhibit high host and tissue specificity, primarily infecting skin and mucosal surfaces. Infection can manifest in a range of ways, from asymptomatic, to the formation of self-resolving papillary masses, to malignant epithelial cancers^12^. This family of viruses has a broad host range, spanning diverse species and environments. Apart from the genus *Alphapapillomavirus* within the subfamily *Secondpapillomavirinae*, which is known to infect fish, all other papillomaviruses belong to the subfamily *Firstpapillomavirinae* and are associated with reptiles, birds, and mammals, from both terrestrial and aquatic environments^13^.

Few papillomaviruses have been identified in pinnipeds. *Z. californianus* papillomavirus (genus *Dyonupapillomavirus*) was reported from California sea lions (*Z. californianus*) presenting with axillary and preputial papillomatous lesions^14^. This virus has been associated with sporadic cases of both *in situ* and invasive squamous cell carcinoma^15^. In addition, various genera of papillomaviruses have been identified in the feces of apparently healthy Weddell seals (*Leptonychotes weddellii*)^16^.

Given the potential for pinniped viruses to cause population-level effects, and the risk posed to veterinarians and rehabilitators from zoonotic diseases, it is crucial to expand our knowledge of seal viruses. Accordingly, we employed traditional veterinary diagnostic techniques in conjunction with metatranscriptomic sequencing of a variety of tissues from seals, with or without clinical or pathological signs of viral infection, to explore the virome of New Zealand fur seals.

## Results

### Overview of metatranscriptomic data

In total, 14 metatranscriptomic libraries were constructed from pools of tissue from 18 individual New Zealand fur seals (library statistics are summarized in Supplementary Table 1). The extracted RNA was pooled according to tissue type. No mammalian-associated viruses were identified from the brain, liver, lung, or kidney. However, a novel papillomavirus was recovered from oral tissue library SL16 and is discussed in further detail below.

### Identification of a novel papillomavirus in a seal with oral papilloma

**Fig. 1:**
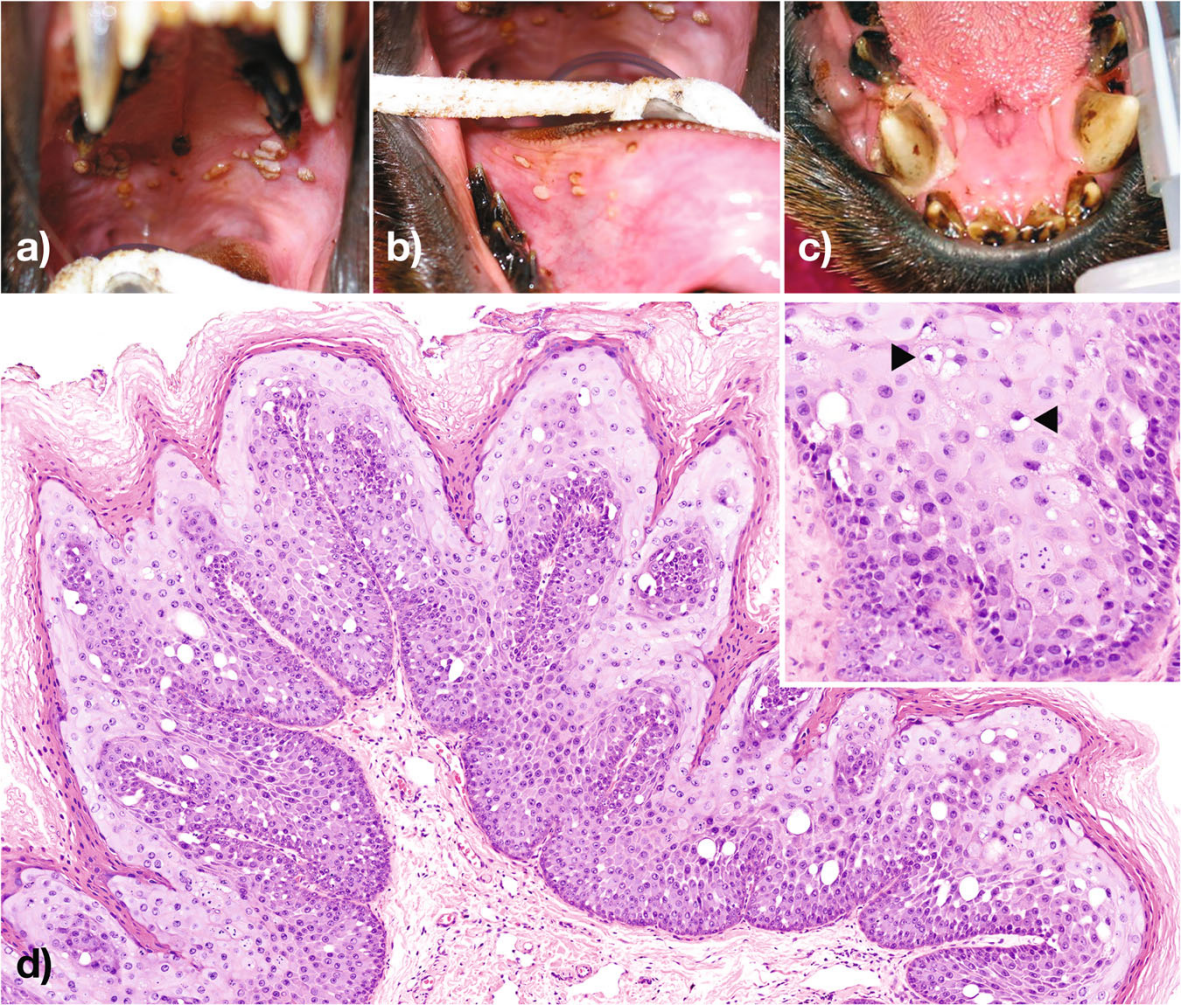
Papillary proliferations, cellular atypia, and the presence of koilocytes in oral lesions. Multifocally coalescing sessile to papillary proliferations of the palatine and lingual epithelium of a New Zealand fur seal Registry #3254 (**a**, **b**). A zone of mucosal pallor surrounding the mandibular canine tooth (**c**). Demarcated lingual epithelial proliferation, with basophilia, and expansion of the stratum spinosum and stratum corneum (**d**—inset higher magnification of the lesion demonstrating cellular atypia and the presence of koilocytes—black arrowheads).

In October 2002, an immature male New Zealand fur seal (Registry #3254) was taken into rehabilitation care after being found hauled out on a beach near Narooma, New South Wales (NSW), Australia, in an emaciated body condition and exhibiting dehydration, severe anemia, and several deep skin wounds over the right hip and right hind flipper, presumably associated with a failed predation attempt by a shark. Upon examination, the seal was noted to have several small raised, sometimes pedunculated and coalescing papillary masses on the roof of the mouth with similar but smaller lesions, evident on the caudoventral right and ventral left aspects of the tongue (Fig. 1a, b). There was also a circumferential zone of mucosal pallor around the right mandibular canine tooth (Fig. 1c). Biopsies of the papillary masses were taken from the roof of the mouth and tongue region in February 2003.

Microscopic examination of multiple formalin-fixed oral lesion biopsies revealed sharply demarcated zones of lingual epithelial proliferation characterized by basophilia, binucleation of cells throughout the stratum basale, and an epithelium irregularly thickened by expansion of the stratum spinosum and stratum corneum (Fig. 1d). Cells in the stratum spinosum were large and often atycal, with large nuclei and abundant pale to basophilic cytoplasm. Epithelial cells with pyknotic or karyorrhectic nuclei, and intercellular oedema were multifocally evident throughout the affected stratum spinosum. Multifocal koilocytes, epithelial cells exhibiting nuclear enlargement and hyperchromasia in conjunction with perinuclear cytoplasmic vacuolation, were also evident within the stratum spinosum. The lingual lamina propria beneath the epithelial lesions appeared normal.

### Papillomavirus genome construction and confirmation

**Fig. 2:**
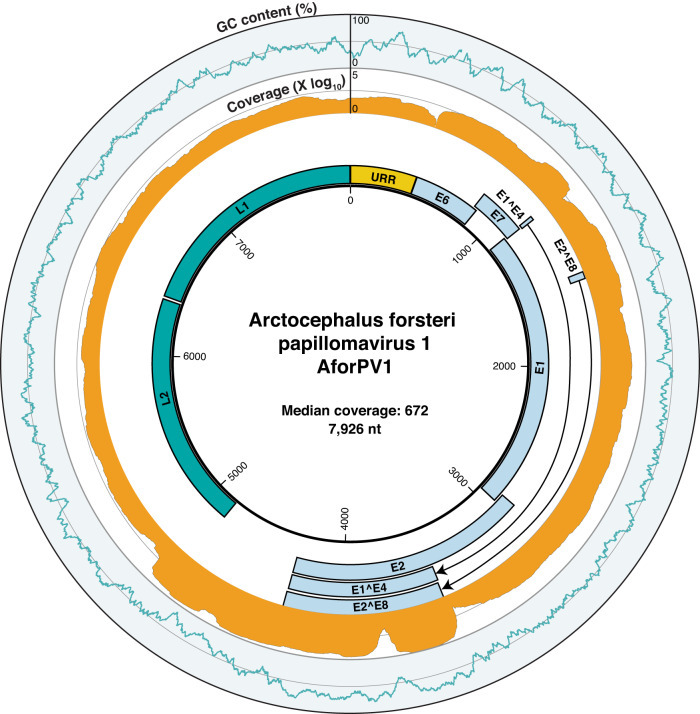
Genome organization of Arctocephalus forsteri papillomavirus 1 (AforPV1). The outer ring indicates the percentage GC content (blue) over a 40 bp sliding window, the middle ring represents read coverage across the genome, log_10_ transformed (orange), and the inner ring illustrates the predicted ORFs and the upstream regulatory region of AforPV1. The “A” in the first start codon of the ORF E6 is assigned as position one.

Several papillomavirus-like contigs were assembled from library SL16. Using a combination of read mapping and RT-PCR, a complete circular genome of 7926 bp here termed *A. forsteri* papillomavirus 1 (AforPV1) was recovered, which was confirmed by CheckV (100% genome completion, high confidence) (Fig. 2). AforPV1 was present in high abundance in SL16 with 0.18% (*n* = 130,197 reads) of total library reads mapping to the genome, slightly less than the abundance of the mitochondrial COX1 gene in this library (0.27%, *n* = 195,218 reads). RT-PCR confirmed that AforPV1 was limited to seal Registry #3254, with both tissue samples taken from under the tongue testing positive. No other tissues or blood samples were available from this individual.

### Evolutionary relationship and genomic properties of AforPV1

**Fig. 3:**
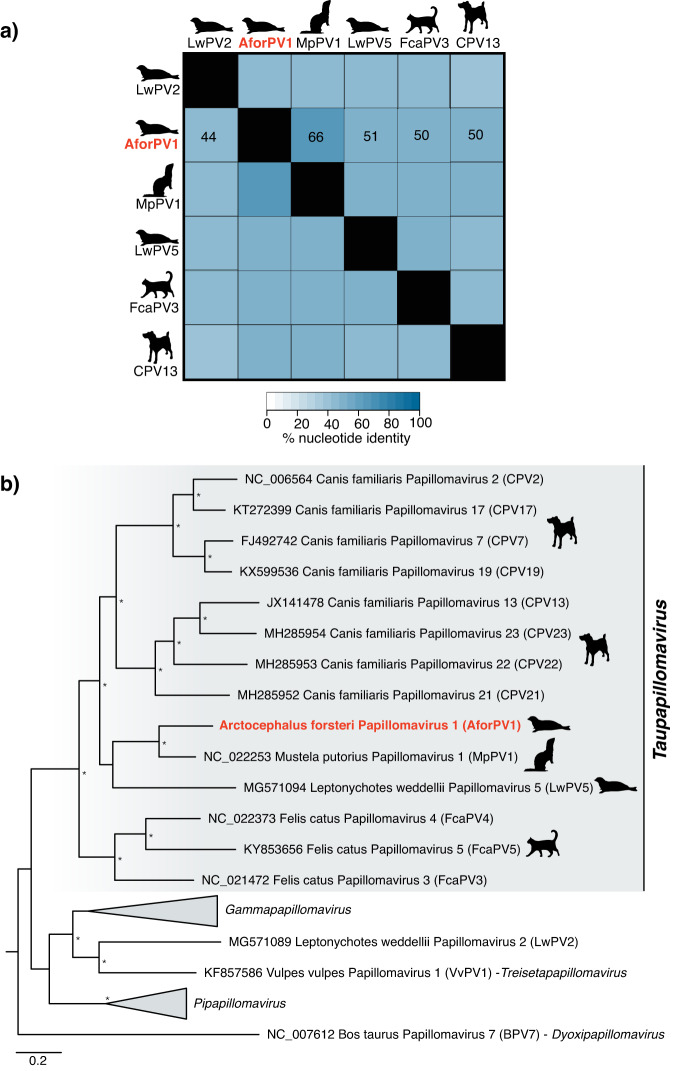
Placement of Arctocephalus forsteri papillomavirus 1 (AforPV1) within the taupapillomaviruses. **a** Percentange identity matrix of representative taupapillomaviruses for each host species compared to AforPV1. **b** Phylogenetic relationship of the taupapillomaviruses and other closely related genera. An ML phylogenetic tree based on the conserved amino acid sequences of the L1, L2, E1, E2 genes shows, in red, the topological position of AforPV1, in the context of its closest relatives. Animal silhouettes depict the virus-host associations for the taupapillomaviruses. All branches are scaled to the number of amino acid substitutions (model LG + F + I + G4) per site, and the tree is midpoint rooted for clarity only. An asterisk indicates node support where SH-aLRT ≥ 80% and UFboot ≥ 95%.

AforPV1 falls as a distinct lineage within the papillomaviruses, with its closest relative Mustela putorius papillomavirus 1 (MpPV1) (NC_022253) isolated from a European polecat (*Mustela putorius*, family *Mustelidae*) with which it shares 66% nucleotide identity across the genome (Fig. 3a). However, the degree of similarity between AforPV1 and MpPV1 varied between the six genes tested (E1, E2, E6, E7, L1 and L2), ranging from 50% (E2) to 74% (L1) nucleotide identity. To determine the evolutionary history of AforPV1 a phylogenetic analysis was conducted using a concatenated alignment of four genes (L1, L2, E1, and E2) as per the ICTV guidelines^13^. This analysis placed AforPV1 within the genus *Taupapillomavirus*, forming a clade with MpPV1 and a papillomavirus associated with the Weddell seal, *L. weddellii* papillomavirus 5 (LwPV5), albeit with poor bootstrap support (SH-aLRT = 43% and UFboot = 64%) (Fig. 3b). Given that the ICTV species demarcation^13^ for the taupapillomaviruses is <70% nucleotide identity across the genome, we suggest that AforPV1 represents a novel species within the genus *Taupapillomavirus* (family *Papillomaviridae*).

The genome organization of AforPV1 is consistent with other taupapillomaviruses, comprising eight open reading frames (ORFs) (E1, E2, E4, E6, E7, E8, L1, and L2) as well as the spliced ORFs of E1^E4 and E8^E2. The presence of the E8^E2 and E1^E4 splice junctions was supported by 89 and 13,700 reads, respectively. No E5 ORF was detected. Six of the eight ORFs had matches to domains associated with the E1, E2, E6, E7, L1, and L2 ORFs (Fig. 2). No domains were detected for the E4 and E8 ORFs. A premature stop codon, which resulted in E2 being 238 bp (79 aa) shorter than its closest relatives, was present. This stop codon was in a region of high coverage and was confirmed by Sanger sequencing. Two zinc-binding sites (CXXC-X29-CXXC) were found in E6, although E6 lacked a PDZ-binding motif (ETQL) in its C-terminus. An alternative pRB-binding site (retinoblastoma tumor suppressor-binding domain) (LXSXE) was detected in E7, consistent with other taupapillomaviruses^16,17^. A cyclin RXL motif (KRRLF) and ATP-binding site (GXXXXGK[T/S]) were detected in E1. The upstream regulatory region (URR), defined as the region between the L1 stop codon and E6 start codon, was 430 bp in length. The URR contained four E2-binding sites (ACCN2-11GGT), one Nf1-binding site (TTGGC), one palindromic E1-binding site (ATTGTTXXXAACAAT) and a TATA box. The predicted ORFs, protein products, and binding sites are shown in Supplementary Table 2.

### Gene expression of AforPV1

Gene expression analysis of AforPV1 revealed variation among its genes (Supplementary Table 3). Notably, E2 displayed relatively high expression levels (194,773 reads per kilobase per million mapped reads [RPKM]). Expression was concentrated in specific regions, encompassing nucleotides 3048–3331 and 3397–3872, which corresponded to the predicted alternatively spliced isoforms, E8^E2 and E1^E4, each recording RPKM values of 315,227 and 315,461, respectively. In contrast, the remaining early genes E1, E6, and E7 exhibited comparatively lower RPKM values of 39,158, 12,894, and 10,751, respectively. The late gene L2 exhibited higher expression (63,078 RPKM) than L1 (4693 RPKM), although this was predominantly observed at the beginning of the L2 gene (nucleotides 4402–4510).

### SL16 library composition

To examine whether other pathogens were present in SL16 (the library in which AforPV1 was found), contigs assembled from rRNA-depleted reads were assessed for taxonomic associations using CCMetagen. SL16 was predominately (85%) comprised of contigs belonging to eared seals (family *Otariidae*), while 5% was attributed to other pinnipeds (e.g., walruses), and non-chordates including bacteria (<0.1%) (Supplementary Fig. 1a). Among the non-chordate abundance, 52% were associated with bacteria from various families, and 48% were linked to eukaryotes, specifically fungi (43%), arthropods (2%), algae (2%), and diatoms (1%) (Supplementary Table 4, Supplementary Fig. 1b). The fungi were primarily assigned to *Candida albicans* (32%).

Further analysis of the CCMetagen results employing BLASTn revealed that a contig with hits to the *Tannerellaceae* (reference sequence *Tannerella forsythia*, HG784150.1) shared 81% nucleotide similarity (*e* value = 7e-93) with the tetratricopeptide repeat protein of *T. forsythia*. The contigs associated with this species were highly fragmented, so we were unable to make a definitive judgment regarding the taxonomy of this bacterium. Additionally, a fragment associated with the *Enterobacteriaceae* (reference sequence *Shigella dysenteriae* EU855235.1) was found to be identical to a hypothetical gene found in *Escherichia coli* (e-value = 0.0, EFO55302.1). The remaining contigs associated with *Enterobacteriaceae* share the greatest sequence similarity with *E. coli* (e-value = 0.0, 95% nucleotide identity, CP017061).

In addition, we identified a partial genome that may represent a novel *Gammaherpesvirus* in a different individual without pathological evidence of viral infection. Information on this putative virus can be found in Supplementary Fig. 2.

## Discussion

We used a metatranscriptomic approach to identify potential viral etiological agents in New Zealand fur seals with and without gross and microscopic pathology. Using this approach on a variety of tissues, we were able to identify novel papillomavirus and a likely etiology for the oral papilloma-like lesions described.

Papillomaviruses have been found in a wide range of mammalian species, with 85 hosts identified to date^18^. Despite this, our understanding of papillomaviruses in seals and their associated pathology remains limited. We identified a novel papillomavirus, the first described in fur seals, and the first report of oral papilloma-like lesions in this host group. Microscopic examination of the oral lesions revealed substantial epithelial cellular atypia and the presence of koilocytes consistent with the cytopathic effects associated with papillomaviruses in humans and other animals^19,20^. Although such lesions are often of limited clinical significance—as observed in this case study where the lesions self-resolved—there is potential for these plaques to progress into invasive squamous cell carcinomas^21^.

A critical factor in papillomavirus-induced oncogenesis is the integration of the virus into the host genome and its impact on the expression of the viral oncogenes E6/E7. These genes are negatively regulated by E2, and integration, which is frequently observed in the HPV^22^ can disrupt the E2 gene, leading to upregulation of the E6/E7 oncogenes^23^. We present preliminary evidence that AforPV1 may exist in both episomal and integrated forms within this individual. The gene expression profile of AforPV1 infection, particularly the high E2/E6 ratio, is typically indicative of episomal infection in HPV16 infection, although exceptions exist^24^. Conversely, the AforPV1 E2 gene appears truncated, suggesting that AforPV1 may also be present in an integrated form. The integration of HPV has been shown to trigger substantial host genome alterations resulting in the complete loss of function^25^ or increased expression in target genes surrounding virus integration sites^26^. Further investigation is required to confirm whether AforPV1 is present in an integrated form and its effect on host gene expression.

While establishing causality is challenging, as papillomaviruses often asymptomatically infect the skin, there are several key pieces of evidence indicating that AforPV1 was infecting the New Zealand fur seal and may be associated with disease in these animals, although further investigation is warranted. AforPV1 appears to be highly abundant within the metatranscriptome data, with SL16 transcripts representing 0.18% of reads in a pool of three individuals, two of which were PCR-negative for this virus. AforPV1 was limited to an individual with papilloma-like lesions, as determined by gross and histological examination prior to the molecular discovery of this virus. This, together with the construction of the entire genome of AforPV1 from metatranscriptomic data, makes it unlikely that this sequence represents an endogenous viral element (i.e. a viral sequence that was passed vertically in the germline of the host) but rather the presence of both integration and episomal AforPV1 replication.

There is no evidence to suggest that another pathogen was responsible for the oral lesions of seal Registry #3254. *C. albicans* was the only microorganism detected in relatively high abundance (0.05% of SL16 abundance in CCMetagen) and is a common oral fungus, acting most commonly as a commensal organism. Although *C. albicans* can be an opportunistic pathogen, it is not known to cause papillary lesions, and these organisms were not evident within multiple histological sections of the lesions^27^. Bacteria were also present in the SL16 library, such as *E. coli* and a sequence with homology to *Tannerella sp*., although in the latter, the highly fragmented nature of the sequences assembled prevented our ability to taxonomically classify this species further. While *E. coli* and certain *Tannerella* species (e.g., *T. forsythia*) are known to be pathogenic, they are not known to cause oral lesions consistent with those described^28,29^.

A phylogenetic analysis provides additional evidence that AforPV1 is indeed associated with a seal host: it groups with the Carnivora-associated taupapillomaviruses in a clade with seal and polecat papillomaviruses, LwPV5 (MG571094)^16^ and MpPV1 (NC_022253)^30^. Although there is some evidence of recombination and host switching within the *Papillomaviridae*, the known taupapillomaviruses appear to have generally co-diverged with their hosts^31^.

The absence of viruses across many of our samples could be attributed to numerous causes. If low abundance viruses are present, it is possible that we did not have the sequencing depth to recover these over the host signal, that viruses are too divergent to detect using similarity-based methods, or that this simply could reflect the absence of infection entirely at the point of sampling. This is supported by the absence of evidence of viral disease in the histopathology reported from these seals.

Through a metatranscriptomic survey of tissue samples from New Zealand fur seals, we identified a novel papillomavirus. The identification of AforPV1 in a seal exhibiting oral papilloma-like lesions emphasizes the value of combining metagenomic sequencing with conventional gross and histological examination to discover novel wildlife pathogens. Such work may ultimately contribute to improved disease detection, control, and conservation efforts. Additional research is necessary to ascertain the clinical relevance of these viruses in New Zealand fur seals.

## Methods

### Sample collection and processing

Samples from live seals were collected by a veterinarian between 2003 and 2021 for diagnostic purposes under a License to Rehabilitate Injured, Sick or Orphaned Protected Wildlife (no. MWL000100542) issued by the New South Wales (NSW) Department of the Environment. Samples from deceased, beach cast, or euthanased seals were collected by the Australian Registry of Wildlife Health (Registry), a conservation science program of Taronga Conservation Society Australia, during routine necropsy for disease surveillance in accordance with NSW National Parks and Wildlife Act 1974, section 132c, Scientific Licence number SL100104. These samples were collected under the auspices of the Taronga Conservation Society Australia’s Opportunistic Sample Policy (approval no. R22D34), and pursuant to NSW Office of Environment and Heritage-issued scientific licenses SL10469 and SL100104. Tissue samples comprising various skin and mucosa samples, liver, brain, lung, and kidney were variably collected from 18 New Zealand fur seals and stored at −80 °C until RNA extraction. A set of tissues representing each organ system was also collected into 10% neutral buffered formalin, embedded in paraffin wax, sectioned and mounted on a glass slide, stained with hematoxylin and eosin, and examined by light microscopy at ×200, ×400, and ×1000 magnification.

### RNA extraction and metatranscriptomic sequencing

Individual tissue aliquots were placed into 600 μl of lysis buffer containing 0.5% foaming reagent (Reagent DX, Qiagen) and 1% β-mercaptoethanol (Sigma-Aldrich), and tissue was homogenized using a TissueRuptor (Qiagen) at a speed of 5000 rpm for up to one minute. The homogenate was centrifuged at maximum speed (15,200 rpm) for three minutes to eliminate any remaining tissue residue. RNA was then extracted from the supernatant using the RNeasy Plus Mini Kit (Qiagen), following the manufacturer’s protocol. Extracted RNA was combined by tissue type into 14 pools with a median of three samples per pool (minimum = 1, maximum = 5) (Supplementary Table 1). Sequencing libraries were constructed using the TruSeq Total RNA Library Preparation Protocol (Illumina). Host ribosomal RNA (rRNA) was depleted using the Ribo-Zero Plus Kit (Illumina), and paired-end sequencing (150 bp) was performed on the NovaSeq 6000 platform (Illumina). Library construction and sequencing were performed by the Australian Genome Research Facility (AGRF).

### Identification of novel virus sequences

Virus identification followed the BatchArtemisSRAMiner pipeline^32^. Briefly, sequencing reads underwent quality trimming and adapter removal using Trimmomatic (v0.38) with parameters SLIDINGWINDOW:4:5, LEADING:5, TRAILING:5, and MINLEN:25, prior to assembly^33^. *De novo* assembly was conducted using MEGAHIT (v1.2.9)^34^. Assembled contigs were compared to the RdRp-scan RdRp core protein sequence database (v0.90)^35^ and the protein version of the Reference Viral Databases (v23.0)^36^ using DIAMOND BLASTx (v2.0.9) with an E-value cut-off of 1 × 10^−5^ ^37^. To exclude potential false positives, contigs with hits to virus sequences were used as a query against the NCBI nucleotide database (as of March 2022) using BLASTn^38^ and the NCBI non-redundant protein (nr) database (as of March 2022) using DIAMOND BLASTx. Using BLASTx and BLASTn matches, virus-like contigs associated with non-vertebrate hosts were excluded.

### *A. forsteri* papillomavirus 1 RT-PCR

RT-PCR was performed on total RNA from the five individual samples that made up library SL16 using primers designed based upon the novel *A. forsteri* papillomavirus 1 (AforPV1) fragments obtained by metatranscriptomic sequencing. SuperScript IV One Step RT-PCR (Invitrogen) and the forward primer 5′-TGGAACGTTGACCTGAGAGA 3’ and reverse primer 5′-AAGGATACGGTCCGTTCTGA-3′ were used to amplify a missing 689 bp section between the L1 and E6 genes. A second set of primers, forward primer 5′-ATACACTCCGTCTTGGGACG-3′ and reverse primer 5′-CAGTTACAAAGCTTCGAGGGT-3′, was used to check the region surrounding the stop codon of E2. The resulting amplicon product was subsequently used for Sanger sequencing at the AGRF.

### Genome analysis and annotation

To examine the genome coverage of each virus, sequence reads were mapped onto virus-like contigs using BBMap (v37.98)^39^, and areas of heterogeneous coverage were manually checked using Geneious (v11.0.9). Where possible, the extremities of contigs were manually extended and re-submitted to read mapping until the contig appeared complete or no overhanging extremities were observed. Sequences of vector origin were detected using VecScreen (https://www.ncbi.nlm.nih.gov/tools/vecscreen/) and removed. GetORF from EMBOSS (v6.6.0) was used to predict open reading frames (ORFs)^40^. To annotate protein functional domains, the InterProScan software package (v5.56) was used with the TIGRFAMs (v15.0), SFLD (v4.0), PANTHER (v15.0), SuperFamily (v1.75), PROSITE (v2022_01), CDD (v3.18), Pfam (v34.0), Hamap (v 2023_01), SMART (v7.1), PRINTS (v42.0), PIRSF (v3.10) and CATH-Gene3D databases (v4.3.0)^41^. The completeness and quality of viral sequences were assessed by visual inspection and the CheckV pipeline^42^. AforPV1 gene expression levels were calculated using htseq-count (v2.0.3) with non-default parameters “-s reverse --nonunique fraction”. AforPV1 binding sites were predicted by manual sequence comparisons, while GC content was calculated in Geneious with a sliding window of 40 nucleotides. AforPV1 spliced ORFs E1^E4 and E8^E2 were manually predicted by aligning AforPV1 to Canis familiaris papillomavirus 19 isolate tvmb1 (KX599536). Support for the splice junctions was assessed using ViReMa (v0.25)^43^. Circos (v0.69-6) was used to produce the circular genome graphs for AforPV1^44^. The marker gene cytochrome c oxidase subunit I (COX1) was identified by querying the contig set against the nr database using DIAMOND BLASTx. The abundances of COX1 and viral transcripts were determined by individually mapping the SL16 reads to each using RNA-Seq by Expectation Maximization (RSEM) software (v1.3.0)^45^.

### Assessment of sequencing library composition

To identify any possible contaminant sequences or coinfecting bacteria or fungi, contigs from the SL16 library were aligned to the custom NCBI nt database using the KMA aligner and the CCMetagen program^46,47^. Species related to known pathogens of mammals were manually confirmed through BLASTn and read mapping against reference genes using BBMap.

### Phylogenetic analysis

Phylogenetic trees of the putative papillomavirus identified here were inferred using a maximum likelihood approach. Representative genomes (*n* = 117) from each of the papillomavirus genera were downloaded from The Papillomavirus Episteme (PaVE) (https://pave.niaid.nih.gov/)^18^. The amino acid sequences of four genes (L1, L2, E1, and E2) were obtained for these sequences along with the novel papillomavirus identified here and individually aligned using MAFFT (v7.402), quality trimmed using trimAl (v1.2), and concatenated to form a single alignment. All phylogenetic trees were estimated using IQ-TREE2^48^. Branch support was calculated using 1000 bootstrap replicates with the UFBoot2 algorithm and an implementation of the SH-like approximate likelihood ratio test within IQ-TREE2^48^. The best-fit model of amino acid substitution was determined using the Akaike information criterion (AIC), the corrected AIC, and the Bayesian information criterion with the ModelFinder function in IQ-TREE2^49^. This process was then repeated with a subset of papillomaviruses, namely the taupapillomaviruses and the related gamma- and pipapillomaviruses.

## Supplementary information

Supplementary Information

Supplementary Table 1

Supplementary Table 2

Supplementary Table 3

Supplementary Table 4

## Data Availability

All *A. forsteri* sequence reads are available on the NCBI Sequence Read Archive (SRA) under BioProject PRJNA1013207. The viral genomes assembled in this study have been deposited in the GenBank and assigned the accession number OR531434, OR590706 and OR590707. The sequences, alignments, and phylogenetic trees generated in this study are available at https://github.com/JonathonMifsud/Identification-of-a-novel-papillomavirus-in-a-New-Zealand-Fur-seal-with-oral-papilloma.

## Code availability

The underlying code for this study is not publicly available but may be made available to qualified researchers on reasonable request from the corresponding author.

## References

[1] Goldsworthy, S. D., Bulman, C., He, X., Larcombe, J. & Littnan, C. Marine mammals: fisheries, tourism and management issues Vol. 2006, 62–99 (CSIRO Publishing Melbourne, 2003).

[2] Shaughnessy, P. D. The action plan for Australian seals. (Environment Australia), (1999).

[3] Bossart, G. D. Marine mammals as sentinel species for oceans and human health. *Vet. Pathol.* **48**, 676–690 (2011). 21160025 10.1177/0300985810388525

[4] Sikorski, A., Dayaram, A. & Varsani, A. Identification of a novel circular DNA virus in New Zealand Fur Seal (*Arctocephalus forsteri*) fecal matter. *Genome Announc.* **1**, e00558–00513 (2013). 23929471 10.1128/genomeA.00558-13 PMC3738887

[5] Dagleish, M. et al. The first report of otarine herpesvirus-1-associated urogenital carcinoma in a South American fur seal (Arctocephalus. australis). *J. Comp. Pathol.* **149**, 119–125 (2013). 10.1016/j.jcpa.2012.10.002 23218410

[6] Deming, A. C. et al. Unlocking the role of a genital herpesvirus, otarine herpesvirus 1, in California Sea lion cervical cancer. *Animals* **11**, 491 (2021). 33668446 10.3390/ani11020491 PMC7918579

[7] Bossart, G. & Duignan, P. Emerging viruses in marine mammals. *CABI Rev.* 1–17 (2019).

[8] Colegrove, K. M., Greig, D. J. & Gulland, F. M. Causes of live strandings of northern elephant seals (*Mirounga angustirostris*) and Pacific harbor seals (*Phoca vitulina*) along the central California coast, 1992-2001. *Aquat. Mamm.* **31**, 1 (2005).

[9] Bodewes, R. et al. Avian Influenza A(H10N7) virus-associated mass deaths among harbor seals. *Emerg. Infect. Dis.* **21**, 720–722 (2015). 25811303 10.3201/eid2104.141675 PMC4378483

[10] Puryear, W. et al. Highly pathogenic avian influenza A(H5N1) virus outbreak in New England Seals, United States. *Emerg. Infect. Dis.* **29**, 786–791 (2023). 36958010 10.3201/eid2904.221538 PMC10045683

[11] Abdelwhab, E. M. & Mettenleiter, T. C. Zoonotic animal influenza virus and potential mixing vessel hosts. *Viruses* **15**, 980 (2023). 37112960 10.3390/v15040980 PMC10145017

[12] Syrjänen, S. Oral manifestations of human papillomavirus infections. *Eur. J. Oral Sci.* **126**, 49–66 (2018). 30178562 10.1111/eos.12538 PMC6174935

[13] Van Doorslaer, K. et al. ICTV virus taxonomy profile: papillomaviridae. *J. Gen. Virol.* **99**, 989–990 (2018). 29927370 10.1099/jgv.0.001105 PMC6171710

[14] Rivera, R. et al. Characterization of a novel papillomavirus species (ZcPV1) from two California sea lions (*Zalophus californianus*). *Vet. Microbiol.* **155**, 257–266 (2012). 22005176 10.1016/j.vetmic.2011.09.027

[15] Luff, J. A., Burns, R. E., Mader, M., Priest, K. D. & Tuttle, A. D. Cutaneous squamous cell carcinoma associated with Zalophus californianus papillomavirus 1 in a California sea lion. *J. Vet. Diagn. Invest.* **30**, 572–575 (2018). 29629648 10.1177/1040638718769702 PMC6471675

[16] Smeele, Z. E. et al. Diverse papillomaviruses identified in Weddell seals. *J. Gen. Virol.* **99**, 549 (2018). 29469687 10.1099/jgv.0.001028 PMC5982131

[17] Wang, J., Zhou, D., Prabhu, A., Schlegel, R. & Yuan, H. The canine papillomavirus and gamma HPV E7 proteins use an alternative domain to bind and destabilize the retinoblastoma protein. *PLoS Pathog.* **6**, e1001089 (2010). 20824099 10.1371/journal.ppat.1001089 PMC2932728

[18] Van Doorslaer, K. et al. The Papillomavirus Episteme: a major update to the papillomavirus sequence database. *Nucleic Acids Res.* **45**, D499–D506 (2017). 28053164 10.1093/nar/gkw879 PMC5210616

[19] Betz, S. J. HPV-related papillary lesions of the oral mucosa: a review. *Head Neck Pathol.* **13**, 80–90 (2019). 30693456 10.1007/s12105-019-01003-7 PMC6405797

[20] Sundberg, J. et al. Feline papillomas and papillomaviruses. *Vet. Pathol.* **37**, 1–10 (2000). 10643975 10.1354/vp.37-1-1

[21] Munday, J. S., Dunowska, M., Laurie, R. E. & Hills, S. Genomic characterisation of canine papillomavirus type 17, a possible rare cause of canine oral squamous cell carcinoma. *Vet. Microbiol.* **182**, 135–140 (2016). 26711040 10.1016/j.vetmic.2015.11.015

[22] Arias-Pulido, H., Peyton, C. L., Joste, N. E., Vargas, H. & Wheeler, C. M. Human papillomavirus type 16 integration in cervical carcinoma in situ and in invasive cervical cancer. *J. Clin. Microbiol.* **44**, 1755–1762 (2006). 16672403 10.1128/JCM.44.5.1755-1762.2006 PMC1479176

[23] Münger, K. et al. Mechanisms of human papillomavirus-induced oncogenesis. *J. Virol.* **78**, 11451–11460 (2004). 15479788 10.1128/JVI.78.21.11451-11460.2004 PMC523272

[24] Qiu, Q. et al. Integrated analysis of virus and host transcriptomes in cervical cancer in Asian and Western populations. *Genomics* **113**, 1554–1564 (2021). 33785400 10.1016/j.ygeno.2021.03.029

[25] Schmitz, M. et al. Loss of gene function as a consequence of human papillomavirus DNA integration. *Int. J. Cancer* **131**, E593–E602 (2012). 22262398 10.1002/ijc.27433

[26] Ojesina, A. I. et al. Landscape of genomic alterations in cervical carcinomas. *Nature* **506**, 371–375 (2014). 24390348 10.1038/nature12881 PMC4161954

[27] Dunn, J. L., Buck, J. D. & Spotte, S. Candidiasis in captive pinnipeds. *J. Am. Vet. Med. Assoc* **185**, 1328–1330 (1984). 6096327

[28] Holt, S. C. & Ebersole, J. L. Porphyromonas gingivalis, Treponema denticola, and Tannerella forsythia: the ‘red complex’, a prototype polybacterial pathogenic consortium in periodontitis. *Periodontol. 2000* **38**, 72–122 (2005). 15853938 10.1111/j.1600-0757.2005.00113.x

[29] Gomes, T. A. et al. Diarrheagenic escherichia coli. *Braz. J. Microbiol.* **47**, 3–30 (2016). 27866935 10.1016/j.bjm.2016.10.015 PMC5156508

[30] Smits, S. L. et al. Metagenomic analysis of the ferret fecal viral flora. *PLoS One* **8**, e71595 (2013). 23977082 10.1371/journal.pone.0071595 PMC3748082

[31] Van Doorslaer, K. Evolution of the *Papillomaviridae*. *Virology* **445**, 11–20 (2013). 23769415 10.1016/j.virol.2013.05.012

[32] Mifsud, J. C. O. BatchArtemisSRAMiner: v1.0.3. Zenodo. 10.5281/zenodo10020164 Available at: https://github.com/JonathonMifsud/BatchArtemisSRAMiner/ (2023)

[33] Bolger, A. M., Lohse, M. & Usadel, B. Trimmomatic: a flexible trimmer for Illumina sequence data. *Bioinformatics* **30**, 2114–2120 (2014). 24695404 10.1093/bioinformatics/btu170 PMC4103590

[34] Li, D., Liu, C.-M., Luo, R., Sadakane, K. & Lam, T.-W. MEGAHIT: an ultra-fast single-node solution for large and complex metagenomics assembly via succinct de Bruijn graph. *Bioinformatics* **31**, 1674–1676 (2015). 25609793 10.1093/bioinformatics/btv033

[35] Charon, J., Buchmann, J. P., Sadiq, S. & Holmes, E. C. RdRp-scan: a bioinformatic resource to identify and annotate divergent RNA viruses in metagenomic sequence data. *Virus Evol.* **8**, veac082 (2022). 36533143 10.1093/ve/veac082 PMC9752661

[36] Goodacre, N., Aljanahi, A., Nandakumar, S., Mikailov, M. & Khan, A. S. A reference viral database (RVDB) to enhance bioinformatics analysis of high-throughput sequencing for novel virus detection. *MSphere* **3**, e00069–00018 (2018). 29564396 10.1128/mSphereDirect.00069-18 PMC5853486

[37] Buchfink, B., Reuter, K. & Drost, H.-G. Sensitive protein alignments at tree-of-life scale using DIAMOND. *Nat. Methods* **18**, 366–368 (2021). 33828273 10.1038/s41592-021-01101-x PMC8026399

[38] Camacho, C. et al. BLAST+: architecture and applications. *BMC Bioinformatics* **10**, 1–9 (2009). 20003500 10.1186/1471-2105-10-421 PMC2803857

[39] Bushnell, B. BBMap: a fast, accurate, splice-aware aligner. https://www.osti.gov/biblio/1241166 (2014).

[40] Rice, P., Longden, I. & Bleasby, A. EMBOSS: the European molecular biology open software suite. *Trends Genet.* **16**, 276–277 (2000). 10827456 10.1016/s0168-9525(00)02024-2

[41] Jones, P. et al. InterProScan 5: genome-scale protein function classification. *Bioinformatics* **30**, 1236–1240 (2014). 24451626 10.1093/bioinformatics/btu031 PMC3998142

[42] Nayfach, S. et al. CheckV assesses the quality and completeness of metagenome-assembled viral genomes. *Nat. Biotechnol.* **39**, 578–585 (2021). 33349699 10.1038/s41587-020-00774-7 PMC8116208

[43] Sotcheff, S. et al. ViReMa: a virus recombination mapper of next-generation sequencing data characterizes diverse recombinant viral nucleic acids. *GigaScience* **12**, giad009 (2023). 36939008 10.1093/gigascience/giad009 PMC10025937

[44] Krzywinski, M. et al. Circos: an information aesthetic for comparative genomics. *Genome Res.* **19**, 1639–1645 (2009). 19541911 10.1101/gr.092759.109 PMC2752132

[45] Li, B. & Dewey, C. N. RSEM: accurate transcript quantification from RNA-Seq data with or without a reference genome. *BMC Bioinformatics* **12**, 323 (2011). 21816040 10.1186/1471-2105-12-323 PMC3163565

[46] Clausen, P. T., Aarestrup, F. M. & Lund, O. Rapid and precise alignment of raw reads against redundant databases with KMA. *BMC Bioinformatics* **19**, 1–8 (2018). 30157759 10.1186/s12859-018-2336-6 PMC6116485

[47] Marcelino, V. R. et al. CCMetagen: comprehensive and accurate identification of eukaryotes and prokaryotes in metagenomic data. *Genome Biol.* **21**, 103 (2020). 32345331 10.1186/s13059-020-02014-2 PMC7189439

[48] Anisimova, M., Gil, M., Dufayard, J.-F., Dessimoz, C. & Gascuel, O. Survey of branch support methods demonstrates accuracy, power, and robustness of fast likelihood-based approximation schemes. *Syst. Biol.* **60**, 685–699 (2011). 21540409 10.1093/sysbio/syr041 PMC3158332

[49] Kalyaanamoorthy, S., Minh, B. Q., Wong, T. K., Von Haeseler, A. & Jermiin, L. S. ModelFinder: fast model selection for accurate phylogenetic estimates. *Nat. Methods* **14**, 587–589 (2017). 28481363 10.1038/nmeth.4285 PMC5453245

