# Supplementary material for: A novel papillomavirus in a New Zealand fur seal (Arctocephalus forsteri) with oral lesions
Source: Npj Viruses. 2024 Mar 21;2:10. doi: 10.1038/s44298-024-00020-w (PMC11721157; doi:10.1038/s44298-024-00020-w)
Supplement: Supplementary file 1 — Supplementary Information [file 44298_2024_20_MOESM1_ESM.pdf]

A novel papillomavirus in a New Zealand fur seal (Arctocephalus forsteri) with oral lesions

Jonathon C.O. Mifsud[1], Jane Hall[2,3], Kate Van Brussel[1], Karrie Rose[3], Rhys H. Parry[4], Edward C. Holmes[1], Erin Harvey[1*]

1. Sydney Institute for Infectious Diseases, School of Medical Sciences, The University of Sydney, Sydney, NSW 2006, Australia.
2. Centre for Planetary Health and Food Security, School of Environment and Science, Griffith University, Nathan, Queensland, Australia
3. Australian Registry of Wildlife Health, Taronga Conservation Society Australia, Mosman, NSW 2088, Australia
4. School of Chemistry and Molecular Biosciences, The University of Queensland, Brisbane, QLD 4067, Australia

Supplementary Information

Contents:

Supplementary Figure 1. Taxonomic assignments of contigs in sequencing libraries

Supplementary Figure 2. Investigation of a partial gammaherpesvirus

The following Supplementary tables are available as .xlsx files:

Supplementary Table 1. Sample collection and library details

Supplementary Table 2. Predicted ORFs, protein products and binding sites

Supplementary Table 3. AforPV1 gene expression analysis

Supplementary Table 4. SL16 metatranscriptome composition

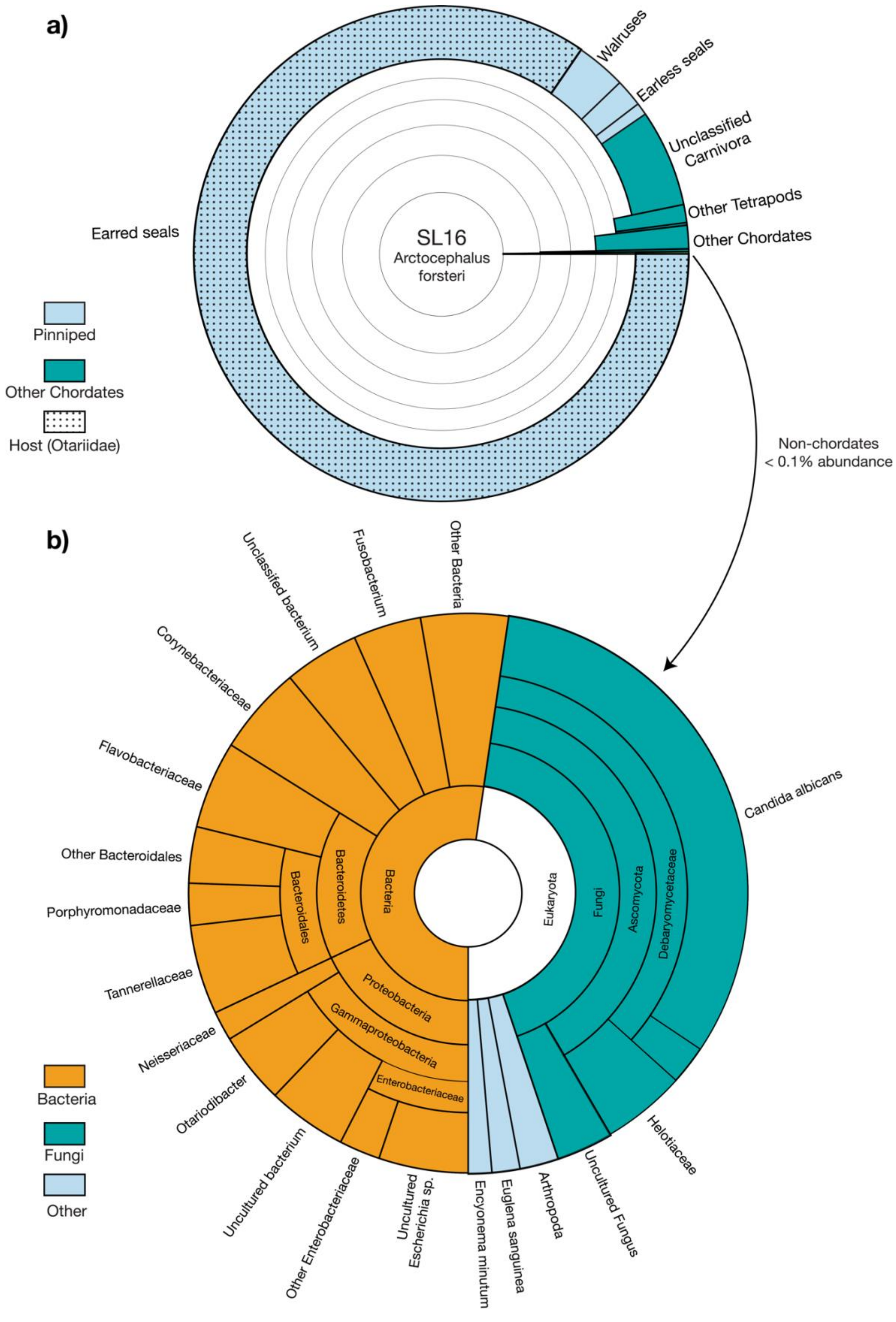

a)
Walruses
Earless seals
Unclassified Carnivora
Other Tetrapods
Other Chordates
Earred seals
SL16
Arctocephalus forsteri
Pinniped
Other Chordates
Host (Otariidae)
Non-chordates
< 0.1% abundance
b)
Unclassifed bacterium
Fusobacterium
Other Bacteria
Corynebacteriaceae
Flavobacteriaceae
Other Bacteroidales
Porphyromonadaceae
Tannerellaceae
Neisseriaceae
Otariodibacter
Uncultured bacterium
Other Enterobacteriaceae
Uncultured Escherichia sp.
Encyonema minutum
Euglena sanguinea
Arthropoda
Uncultured Fungus
Helotiaceae
Candida albicans
Bacteria
Bacteroidetes
Bacteroidales
Proteobacteria
Gammaproteobacteria
Enterobacteriaceae
Eukaryota
Fungi
Ascomycota
Debaryomycetaceae
Bacteria
Fungi
Other

Supplementary Figure 1. Taxonomic assignments of contigs in sequencing libraries (A) Krona graphs illustrating the relative abundance of taxa in the SL16 metatranscriptome at varying taxonomic levels. For clarity, a maximum depth of six taxonomic levels was chosen. (B) Krona graph of the non-chordate subset of taxa. Across both panels segments are highlighted based on the species' taxonomic grouping. Dots have been used to signify where contigs have been taxonomically assigned within the same family (*Otariidae*) as the New Zealand fur seal (*Arctocephalus forsteri*). Contigs without any matches in the database are not shown.

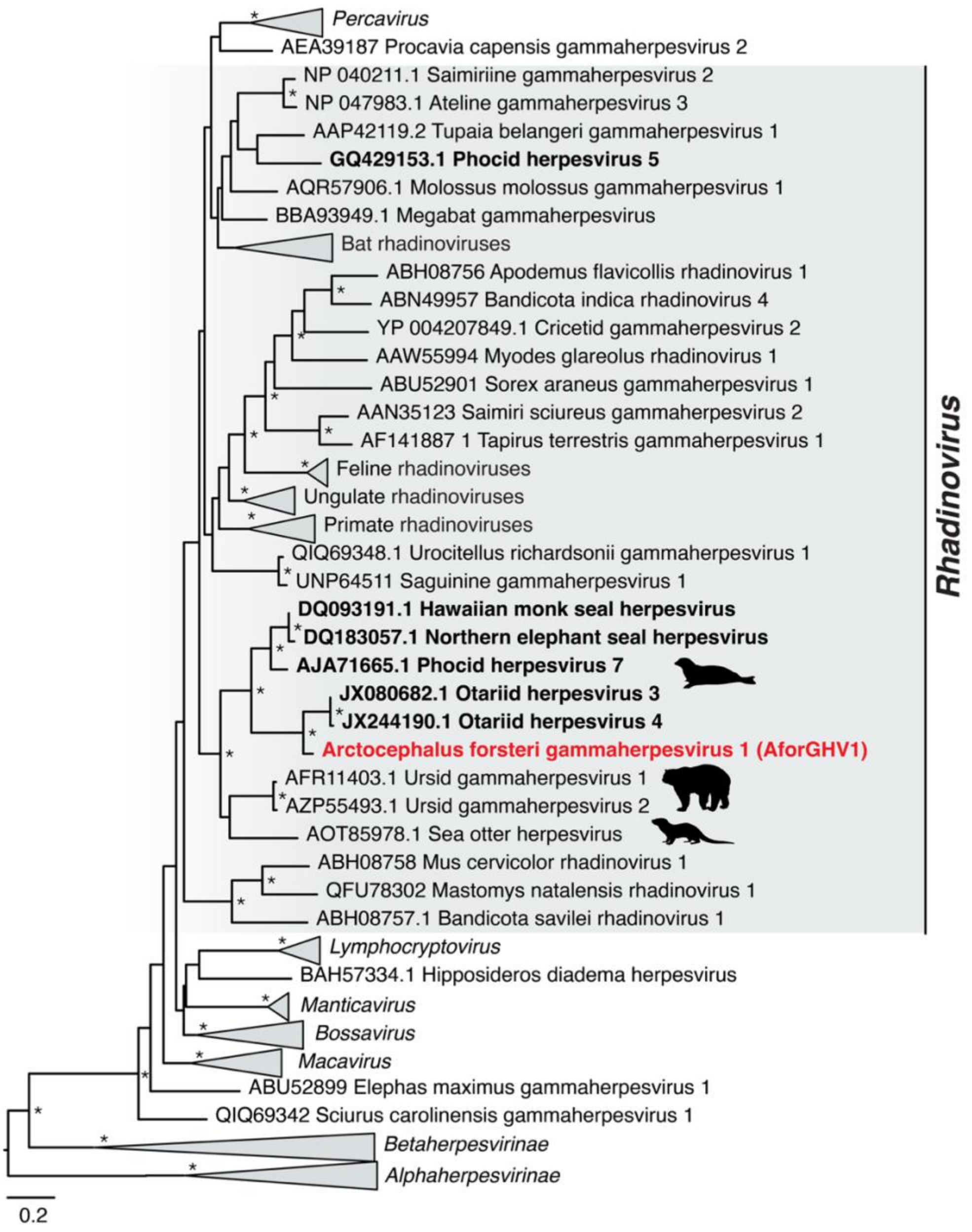

Supplementary Figure 2. Investigation of a partial gammaherpesvirus

Phylogenetic relationships of the *Herpesviridae*. An ML phylogenetic tree based on the conserved amino acid sequences of the DNA polymerase gene with Arctocephalus forsteri gammaherpesvirus 1 (AforGHV1) shown in red and in the context of its closest relatives. The tip labels of pinniped associated herpesviruses are bolded. Animal silhouettes depict the virus-host associations for the rhadinoviruses. All branches are scaled to the number of amino acid substitutions (model LG+F+R9) per site, and the tree is midpoint rooted for clarity only. An asterisk indicates node support where SH-aLRT >= 80% and UFboot >= 95%.

Investigation of a partial gammaherpesvirus

In the SL16 library, several fragmented contigs exhibited similarities to gammaherpesviruses, comprising 0.005% of the total reads (n = 3,503). RT-PCR analysis confirmed that this sequence was exclusive to the oral tissue of seal Registry #10741, with no presence in the genital tissue of this individual. This seal was an immature male rescued at Cronulla, NSW, Australia, in September 2015 presenting in an emaciated body condition with various traumatic injuries and was euthanased due to a poor prognosis for recovery. Gross and histopathological examinations confirmed the animal's emaciated state, the presence of intestinal helminths (within the expected range for a free-ranging pinniped), severe traumatic injuries, and a neutrophilia consistent with a systemic inflammatory response secondary to infected wounds. Genital and oral mucosal tissue samples were taken from this individual for RNA extraction and pooled to form library SL16, the same library in which the papillomavirus contigs were identified.

Two contigs containing a partial polymerase gene (3,460 bp) and the major capsid protein (12,493 bp) were assembled from the RNA libraries. In an effort to recover more of the herpesvirus genome we used a viral particle enrichment protocol [1] designed to isolate and randomly amplify both DNA and RNA from the oral tissue of a juvenile male seal TARZ-10741. Briefly, tissue was homogenised, filtered, centrifuged, and nuclease treated prior to viral nucleic acid extraction with the QIAamp viral RNA mini kit (Qiagen) [1,2]. Following this, nucleic acids were randomly amplified using the Whole Transcriptome Amplification kit (WTA2, Sigma Aldrich) with modifications [1], and purified using the GenElute PCR cleanup kit (Sigma Aldrich). The DNA library was prepared using the Illumina DNA M preparation kit and sequenced on the Illumina NovaSeq 6000 platform at the AGRF. As the standard clean-up protocol depletes amplicons < 500 bp, these amplicons underwent Illumina purification using beads at a ratio of 1.8x volume to supernatant.

DNA viral particle enrichment yielded related reads (6,310 reads when mapped to NC_035117, the closest relative with a 60% nucleotide identity cut-off), but we were unable to substantially extend the contigs recovered through RNA-seq or recover other core gammaherpesvirus genes. The partial polymerase sequence shared 72% amino acid identity with phocid herpesvirus 7 (AJA71665.1). Phylogenetic analysis revealed that this sequence grouped within a clade of pinniped-associated gammaherpesviruses within the genus *Rhadinovirus* (S2 Figure 1). We tentatively assign this virus as Arctocephalus forsteri gammaherpesvirus 1 (AforGHV1). Both AforGHV1 fragments have been deposited in GenBank and assigned accession numbers OR590706 and OR590707. Further work is undoubtedly needed to characterise this virus and to understand the presence and impact of gammaherpesviruses in New Zealand fur seals.

Supplementary References

1 Conceição-Neto, N. *et al.* Modular approach to customise sample preparation procedures for viral metagenomics: a reproducible protocol for virome analysis. *Sci. Rep.* **5**, 16532, doi:10.1038/srep16532 (2015).

2 Chong, R. *et al.* Fecal Viral Diversity of Captive and Wild Tasmanian Devils Characterized Using Virion-Enriched Metagenomics and Metatranscriptomics. *J. Virol.* **93**, e00205-00219, doi:10.1128/jvi.00205-19 (2019).
